# Association of angiotensin converting enzyme inhibitors and angiotensin II receptor blockers with risk of COVID‐19, inflammation level, severity, and death in patients with COVID‐19: A rapid systematic review and meta‐analysis

**DOI:** 10.1002/clc.23421

**Published:** 2020-08-05

**Authors:** Xiao Liu, Chuyan Long, Qinmei Xiong, Chen Chen, Jianyong Ma, Yuhao Su, Kui Hong

**Affiliations:** ^1^ Department of cardiovascular medicine The Second Affiliated Hospital of Nanchang University Nanchang Jiangxi China; ^2^ Jiangxi Key Laboratory of Molecular Medicine Nanchang Jiangxi China

**Keywords:** ACEI/ARB, COVID‐19, hypertension, infectious disease, lung, pneumonia, SARS‐COV‐2

## Abstract

An association among the use of angiotensin converting enzyme (ACE) inhibitors and angiotensin‐receptor blockers (ARBs) with the clinical outcomes of coronavirus disease 2019 (COVID‐19) is unclear. PubMed, EMBASE, MedRxiv, and BioRxiv were searched for relevant studies that assessed the association between application of ACEI/ARB and risk of COVID‐19, inflammation level, severity COVID‐19 infection, and death in patients with COVID‐19. Ten studies were included with 13,944 patients. ACEI/ARB therapy might be associated with the reduced inflammatory factor (interleukin‐6) and elevated immune cells counts (CD3, CD8). Meta‐analysis showed no significant increase in the risk of COVID‐19 infection (odds ratio [OR]: 0.95, 95% CI: 0.89‐1.05) in patients receiving ACEI/ARB therapy, and ACEI/ARB therapy was associated with a decreased risk of severe COVID‐19 (OR: 0.75, 95% CI: 0.59‐0.96, *p* = 0.02) and mortality (OR: 0.57, 95% CI: 0.37‐0.87, *p* = 0.009). Subgroup analyses showed among the general population, ACEI/ARB therapy was not associated with reduced risks of severe COVID‐19 infection (OR: 0.85, 95% CI: 0.66‐1.08, *p* = 0.19) and all‐cause mortality (OR: 0.31, 95% CI: 0.13‐0.75), and COVID‐19 infection (OR: 0.97, 95% CI: 0.89‐1.05, *p* = 0.45) were not increased. Among patients with hypertension, the use of an ACEI/ARB was associated with a non‐significant lower severity of COVID‐19 (OR: 0.73, 95% CI: 0.51‐1.03, *p* = 0.07) and significant lower mortality (OR: 0.57, 95% CI: 0.37‐0.87, *p* = 0.009), without evidence of an increased risk of COVID‐19 infection (OR: 1.00, 95% CI: 0.90‐1.12, *p* = 1.00). On the basis of the available evidence, ACEI/ARB therapy should be continued in patients who are at risk for, or have COVID‐19, either in general population or hypertension patients. Our results need to be interpreted with caution considering the potential for residual confounders, and more well‐designed studies that control the clinical confounders are necessary to confirm our findings.

## 1 INTRODUCTION

The coronavirus disease 2019 (COVID‐19) pandemic is becoming one of the most far‐reaching public health crises in recorded history. At right time of writing this review, on 29 April 2020, the number of infected persons worldwide has exceeded 3.01 million, with more than 200 000 reported deaths (http://2019ncov.chinacdc.cn/2019-nCoV/global.html). At present, there is no specifically targeted, effective treatment for patients with COVID‐19. There is an urgent need, therefore, to determine how to alleviate the severe clinical symptoms and reduce the morbidity and mortality due to this disease. Severe acute respiratory syndrome coronavirus 2 (SARS‐CoV‐2) shares the same cell entry receptor as SARS‐CoV, in which the viruses' spike proteins bind to the host cell surface. Angiotensin converting enzyme 2 (ACE2) receptors, which are an essential regulator of renin‐angiotensin‐aldosterone system (RAAS) activity.^1^, ^2^ The RAAS is a vital regulator of cardiovascular and renal function, including blood pressure, and which plays a vital role in regulating acute lung injury.^3^, ^4^ The ACE2 level is significantly reduced in patients following SARS‐CoV infection, resulting in RAAS system imbalance, and eventually cause severe acute lung injury. Angiotensin I converting enzyme inhibitors (ACEIs) and Angiotensin II receptor blockers (ARBs) are ACE2 receptor antagonists that can reduce the activity of the RAAS system and which have been widely used in the past several decades to treat cardiovascular diseases, such as hypertension and hypertrophic cardiomyopathy.^3^ However, some animal experiments and clinical trials have shown that ACEIs potentially result in an increase in ACE2 receptors.^5^ Some concern has therefore been expressed that the use of ACEIs/ARBs might increase the risk of COVID‐19 infection after exposure to SARS‐CoV‐2 and result in a poor prognosis.^6^ On 12 April 2020, the European Society of Hypertension COVID‐19 Task Force issued the following statement: the current evidence does not support that RAAS inhibitors aggravate the condition of COVID‐19 patients.^5^

Accumulating evidence revealed that inflammatory cytokine storm and dysfunction of immune system largely contribute to the severe COVID‐19, even cause death.^7^ The latest evidence shows that peripheral blood interlekin‐6 (IL‐6) levels, a critical mediator of respiratory failure, shock, and multiorgan dysfunction, were significantly increased in COVID‐19 patients who had used a RAAS inhibitor.^8^, ^9^ Moreover, CD3 and CD8 cell counts were significantly reduced, suggesting that the level of inflammation in patients treated with ACEIs is relatively low.^8^ Several retrospective studies have shown that the use of ACEIs/ARBs prior to COVID‐19 infection could reduce the severity of COVID‐19 and was associated with a better prognosis, especially in patients with both hypertension and COVID‐19.^10^, ^11^, ^12^ However, other studies have shown that there is no significant correlation between the use of ACEIs/ARBs and the severity and mortality of patients with COVID‐19.^10^, ^12^, ^13^, ^14^, ^15^, ^16^ Because the affection of RAAS inhibitors on the clinical prognosis of COVID‐19 patients is unclear; in this article, we will review the current evidence and assess the clinical prognosis of COVID‐19 patients with or without hypertension treated with ACEs/ARBs.

## 2 METHODS

This study was performed according to PRISMA guidelines (http://www.prisma-statement.org; Table S1).^17^

### 2.1 Literature search

Two authors (L. X. and L. C‐Y.) independently searched the PubMed and Embase databases for published articles and the preprint platforms medRxiv (https://www.medrxiv.org/) and bioRxiv (https://www.biorxiv.org/) (since many studies are available on these websites prior to publication, which allows for collection of the latest data) without language restrictions. Furthermore, the research references were traced and cross‐checked. In the case of any discrepancy, it was resolved by consensus with the third author (H. K.). The databases were searched for articles from 1 February 2019 to 1 May 2020 using the following search terms: ACE inhibitor, angiotensin II receptor blocker, angiotensin‐converting enzyme inhibitor, 2019‐novel coronavirus, SARS‐CoV‐2, COVID‐19, and 2019‐nCoV.

### 2.2 Study selection

Studies were considered eligible for inclusion if they (a) were designed as a randomized controlled trial, case‐control study, or cohort study; and (b) assessed the relationship between ACEI/ARB use and the level of inflammation, disease severity, and mortality in patients with COVID‐19. If multiple studies used the same population, we selected the most recent publication. Certain publication types (eg, reviews, editorials, letters, conference abstracts, and animal studies) or studies with insufficient data were excluded from this analysis.

### 2.3 Data extraction and quality assessment

Two authors (L. X. and L. C‐Y.) independently extracted the study information and the basic characteristics of the articles using a standardized form, including the first author, year of publication, country, sample size, sex ratio, age, sample size, RAAS type, length of follow‐up, adjustments for confounders and adjusted odds ratio (OR) with 95% confidence intervals (CIs). Two authors independently used modified Jadad scores and Newcastle‐Ottawa Scale (NOS) scores to evaluate the quality of the randomized controlled trials (RCTs) and observational studies, respectively. A Jadad score >5 and an NOS score >7 were considered high quality scores.^19^

### 2.4 Statistical analyses

The RevMan5.3 (Review Manager [RevMan], version 5.3, Cochrane Collaboration) software was used for statistical data processing, and the OR and 95% CIs were used to estimate the effect. Heterogeneity among the studies was analyzed using the *I* ^2^ test with the following interpretation: low heterogeneity, defined as *I* ^2^ < 50%; moderate heterogeneity, defined as *I* ^*2*^ = 50% to 75%; and high heterogeneity, defined as *I* ^*2*^ > 75%.^20^ The meta‐analysis was performed using the random effects model when the heterogeneity was more than 25%, otherwise, the fixed effects model was applied. If there were ACEI and ARB subgroups in the study, the combined OR and the 95% CIs were pooled using the RevMan5.3 software. If the study did not report the OR directly, count data for outcomes were used to generate unadjusted ORs and 95% CIs. We also excluded reports with unadjusted ORs in the sensitivity analysis to assess the robustness of our results. A funnel plot was used to test for the presence of publication bias, and *P* value <.05 was considered statistically significant.

## 3 RESULTS

### 3.1 Study selection

**Figure 1:**
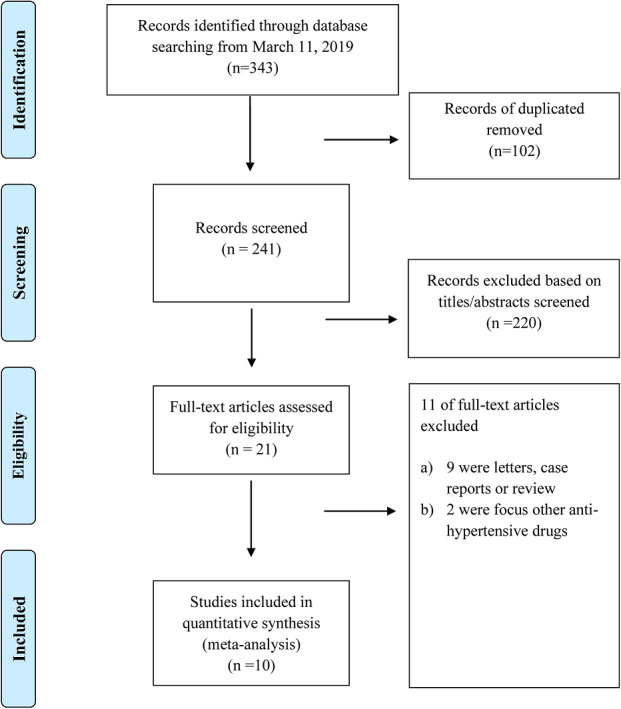
PRISMA flow diagram

The systematic search of the electronic databases identified 343 articles (PubMed = 54, EMBASE = 112, Medrxiv = 132, ArXiv = 45). After excluding duplicates and title/abstracts screened, 21 articles underwent a more detailed full‐text assessment, after which a total of 10 articles with 13,944 patients were included^8^, ^10^, ^11^, ^12^, ^13^, ^14^, ^15^, ^16^, ^21^, ^22^ (Figure 1).

### 3.2 Study characteristics and quality

Table 1 shows the basic characteristics of the included studies. Overall, the sample sizes included in the articles ranged from 42 to 6,272, and ages ranged from 55 to 67 years old. Among the 10 studies, 6 were published^8^, ^10^, ^14^, ^15^, ^16^, ^22^ and 4 were found on a preprint server^11^, ^12^, ^13^, ^21^; 2 studies were based on the general population of COVID‐19 patients^11^, ^14^ and 8 were based on patients with COVID‐19 and hypertension.^8^, ^10^, ^12^, ^13^, ^15^, ^16^, ^21^, ^22^ Four articles reported the effect of ACEIs/ARBs on the level of inflammation,^8^, ^10^, ^12^, ^16^ two studies assessed the risk of COVID‐19 inflection,^14^, ^15^ and all included studies evaluated the severity of disease or/and mortality.^8^, ^10^, ^11^, ^12^, ^13^, ^14^, ^15^, ^16^, ^21^, ^22^ All included studies were observational studies. The NOS score of all of the observational studies was >6, indicating that all of the studies were of high quality (Table S2).

**Table 1:** General characteristics of the included studies in the meta‐analysis

References(first author, year, country/region)	Type of study	Study participants	Sample size	Hypertension%	Age (years), Male	Duration of follow‐up*	Outcomes reported	Adjustments for confounders
Bean, 2020, United Kingdom	RC	General population	205	51.2%	63.0,51.7%	Within 7 days	Severity of COVID‐19	Age, sex, hypertension, diabetes mellitus, ischaemic heart disease, heart failure
Li,2020, China	RC	Hypertension	1,178	100%	55.5,46.3%	Median 19 days	Level of inflammatory cytokines Severity of COVID‐19Death	NA
Mancia, 2020, Italy	Case‐control	General population	6,272	Na	68,63.3%	NA	Risk of COVID‐19Severity of COVID‐19	CCB, diuretics, oral antidiabetic drugs, cardiovascular disease, respiratory diseases, kidney disease, cancer
Liu,2020, China	RC	Hypertension	511	100%	65.2,55.1%	NA	Severity of COVID‐19	NA
Meng, 2020, China	RC	Hypertension	42	100%	64.5,57.1%	NA	Level of inflammatory cytokines Severity of COVID‐19	NA
Yang, 2020, China	RC	Hypertension	126	100%	66.0,49.2%	Mean 30 day	Severity of COVID‐19Level of inflammatory cytokinesDeath	NA
Zeng, 2020, China	RC	Hypertension	75	100%	67.0,55%	28‐days	Severity of COVID‐19Level of inflammatory cytokines	NA
Huang, 2020, China	RC	Hypertension	50	100%	Na,54.0%	Mean 42.3 days	Death	NA
Zhang, 2020, China	RC	Hypertension	1,128	100%	63.4,53.2%	28‐days	Death	Age, gender, fever, cough, dyspnea, diabetes, coronary heart disease, and chronic renal disease, CT‐diagnosed bilateral lung lesions, and incidence of increased CRP and creatinine, D‐dimer, procalcitonin, and unilateral lesion and antiviral drug and lipid lowering drug
Reynolds, 2020, United Status	RC	Hypertension	4,357	100%	64,50.8%	NA	Risk of COVID‐19Severity of COVID‐19	Age; sex; race; ethnic group; body‐mass index; smoking history; history of hypertension, myocardial infarction, heart failure, diabetes, chronic kidney disease, and obstructive lung disease (e.g., asthma and obstructive pulmonary diseases); and other classes of medication

Abbreviations: CCB: Calcium‐channel blockers, COVID‐19: Coronavirus disease 2019, NA: Not available, RC: Retrospective cohort. * the period of time for all‐cause mortality observed.

### 3.3 Inflammation level

There were four articles that evaluated the relationship between ACEI/ARB and level of inflammation in patients with COVID‐19,^8^, ^13^, ^17^, ^22^ but since the data were not reported in the same unit across studies, they were not pooled; therefore, we conducted a systematic review instead (Table 2 **)**. Meng et al.^8^ first assessed the impact of RAAS inhibitors on the level of inflammation in patients with COVID‐19, and although they found no significant change in C‐reactive protein (CRP) levels from the peripheral blood in patients receiving ACEI/ARB treatment, IL‐6 level had a trend of decrease (no statically significance difference may limited by small sample size), and the immune cells (CD3, CD8 T cells) counts were significantly increased.

**Table 2:** Effect of ACEI/ARB on level of inflammatory cytokines in patients with COVID‐19

References (first author, year, country/region)	Study participants	Sample size	Effect on inflammatory cytokines	Values (median [IQR])		
				ACEI/ARB	Non‐ACEI/ARB	*P*
Li, 2020, China [16]	Hypertension	1178	Interleukin 6 **↓**, pg/mL C‐reactive protein**→**, mg/dL	7.5 (3.3‐22.2) 2.1 (0.3‐5.2)	8.8 (4.1‐30.8) 2.6 (0.4‐6.0)	.06 .99
Meng, 2020, China [8]	Hypertension	42	Interleukin 6 **↓** ^a^ C‐reactive protein **→** CD3 cell count**↑** CD4 cell count **→** CD8 cell count**↑**	Na	Na	Na
Yang, 2020, China [12]	Hypertension	126	Interleukin 6 **→** C‐reactive protein↓	14.3 (3.7‐121.1) 11.5 (4.0‐58.2)	10.1 (5.0‐50.4) 33.9 (5.1‐119.2)	.52 .049
Zhang, 2020, China [10]	Hypertension	1128	C‐reactive protein^b^ **→**	74/124 (59.7)	136/221 (61.5)	.73

Abbreviations: ACEI/ARB: angiotensin I converting enzyme inhibitors/angiotensin II receptor blockers; CT: computed tomography; Na: not available.

^a^ A trend of decrease, but no statically significance difference may limited by small sample size.

^b^ Values expressed as unit of increase> upper limit of normal, n/N (%); IQR: interquartile range; CD3.

In contrast, another study showed that there was no difference in IL‐6 levels, while high‐sensitivity CRP test was significantly decreased.^13^ A study that included 342 COVID‐19 patients with hypertension who had taken ACEI/ARB drugs found that IL‐6 levels were significantly reduced, while CRP level shows no significant difference.^17^ More recently, a multicenter study including 1128 patients with hypertension and COVID‐19 reported that the number of patients with high CRP values who had received ACEI/ARB therapy showed no significantly different than in the patients who had not received ACEI/ARB therapy.^10^

### 3.4 Risk of COVID‐19 infection

**Figure 2:**
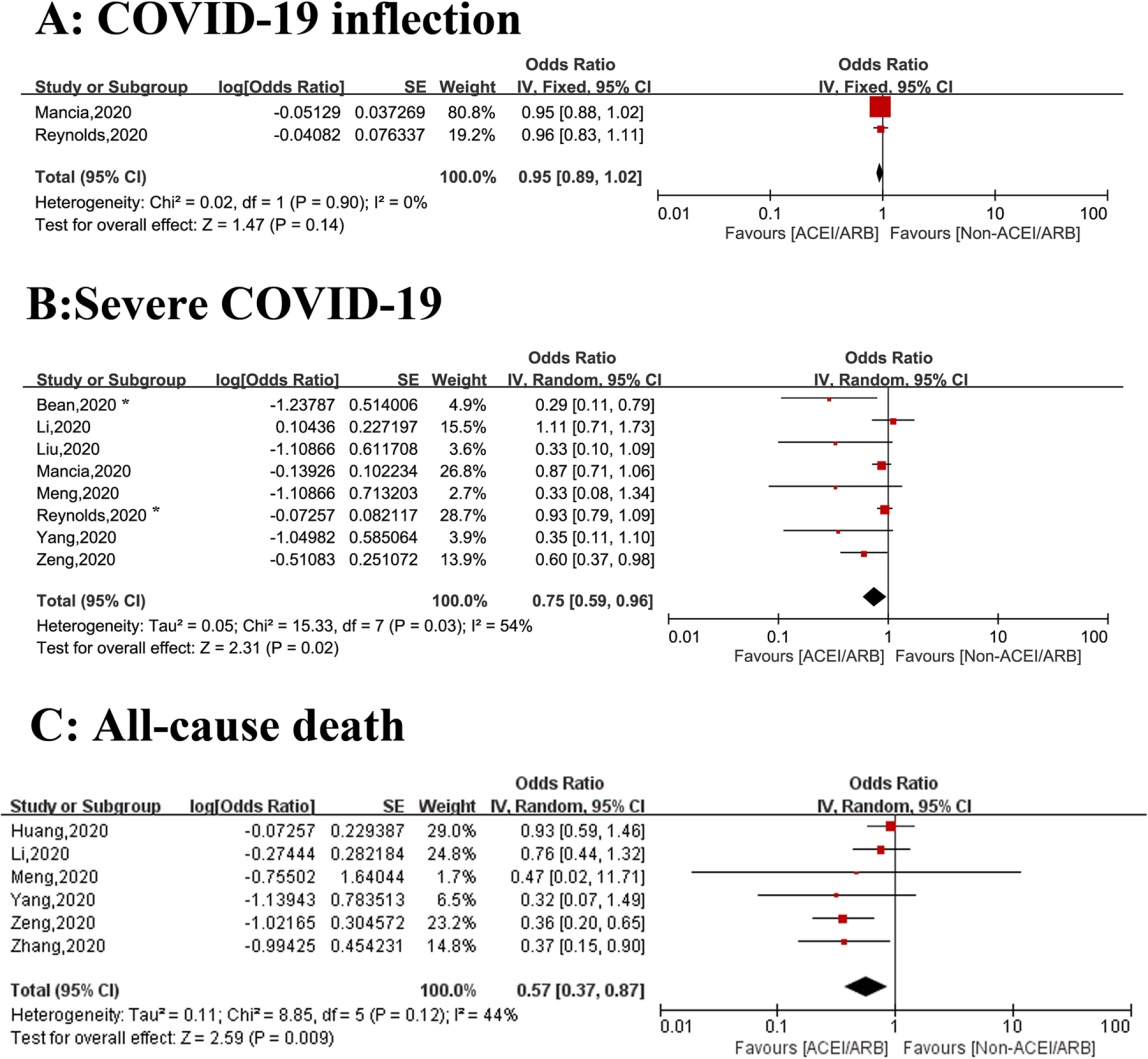
Summary of the associations between use of ACEI/ARB and clinical outcomes among patients with COVID‐19. A, Risk of COVID‐19 infection. B, Risk of severe COVID‐19 infection. C, All‐cause death. *severe COVID‐19 or death. ACEI, angiotensin I converting enzyme inhibitor; ARB, angiotensin II receptor blockers; COVID‐19, coronavirus disease 2019

**Figure 3:**
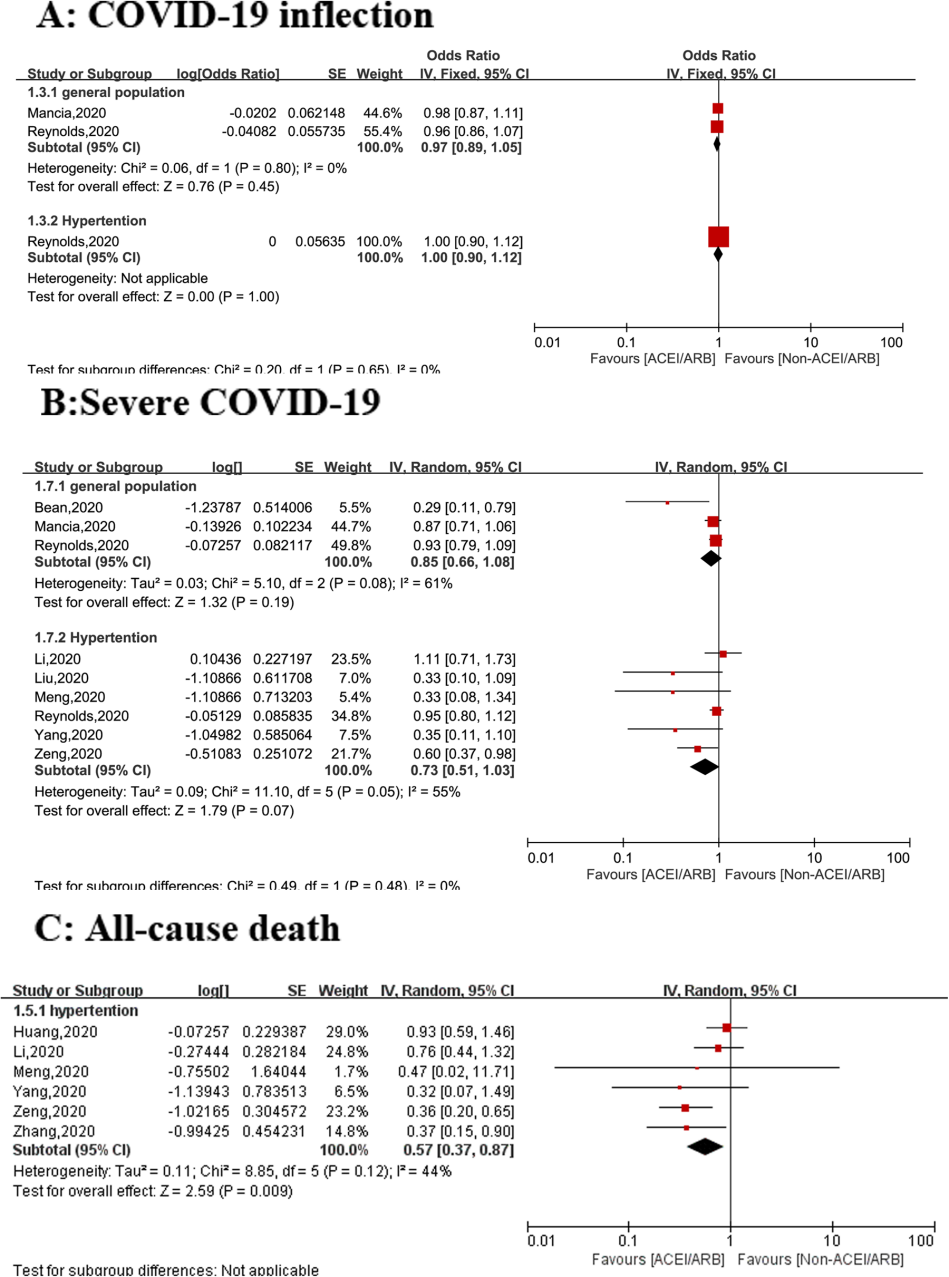
Subgroup analysis of the associations between use of ACEI/ARB and clinical outcomes among patients with COVID‐19 stratified by general population and hypertensive population: A, Risk of COVID‐19 infection. B, Risk of severe COVID‐19 infection. C, All‐cause death. *severe COVID‐19 or death. ACEI, angiotensin I converting enzyme inhibitor; ARB, angiotensin II receptor blockers; COVID‐19, coronavirus disease 2019

Two^15^, ^16^ studies including a total of 10 629 patients reviewed the risk of COVID‐19 infection, including one general population‐based study and one study including patients with hypertension. There was no significant increase in the risk of COVID‐19 infection in patients receiving ACEI/ARB therapy (OR = 0.95, 95% CI: 0.89‐1.02; *P* = .14, *I* ^2^ = 0%), with no significant heterogeneity (Figure 2A). Subgroup analysis based on population showed that the results did not change in the general population (OR = 0.96, 95% CI: 0.89‐1.05; *P* = .45, *I* ^2^ = 0%) or in the hypertensive population (OR = 1.00, 95% CI: 0.90‐1.12; *P* = 1.00) (Figure 3A).

### 3.5 Risk of severe COVID‐19

Eight studies involving 12,766 patients assessed the relationship between the use of ACEI/ARB therapy and severe COVID‐19,^8^, ^11^, ^12^, ^13^, ^14^, ^15^, ^16^, ^21^ with two reports in the general population,^11^, ^14^ and six reports in patients with hypertension.^8^, ^12^, ^13^, ^15^, ^16^, ^21^ Compared with the non‐ACEI/ARB group, the risk of severe COVID‐19 infection decreased by 35% (OR = 0.75, 95% CI: 0.59‐0.96; *P* = .02) in patients treated with an ACEI/ARB, with moderate heterogeneity (*I* ^2^ = 54%) (Figure 2B). However, in the sensitivity analysis, the results were not statistically significant when the unadjusted studies^8^, ^12^, ^13^, ^16^, ^21^ were excluded (OR = 0.85, 95% CI: 0.66‐1.08; *P* = .19).

Subgroup analysis showed that there was no statistical significant association between the risk of severe COVID‐19 infection and ACEI/ARB use either in the general population (OR = 0.85, 95% CI: 0.66‐1.08; *P* = .19, *I* ^2^ = 61%) or in hypertensive patients with (OR = 0.73, 95% CI: 0.51‐1.03; *P* = .07, *I* ^2^ = 55%) (Figure 3B).

### 3.6 All‐cause mortality

Six studies reported all‐cause mortality in 2,599 patients with COVID‐19,^8^, ^10^, ^12^, ^16^, ^21^, ^22^ and ACEI/ARB therapy was associated with a decreased risk of all‐cause mortality (OR: 0.57, 95% CI: 0.37‐0.87; *P* = 0.009) with modest of heterogeneity (*I* ^2^ = 44%) (Figure 2C). There is no evidence of heterogeneity (*I* ^2^ = 3%) when one^22^ study was excluded, with no change of the conclusion (OR: 0.49, 95% CI: 0.34‐0.71; *P* = 0.0001). In the sensitivity analysis, the results were statistically significant when the unadjusted studies^8^, ^12^, ^16^, ^21^, ^22^ were excluded (OR = 0.37, 95% CI: 0.15‐0.90; *P* = 0.03).

Subgroup analysis of hypertensive patients^8^, ^10^, ^12^, ^16^, ^21^, ^22^ (N = 2,599) showed the use of an ACEI/ARB is associated with a decreased risk of all‐cause mortality (OR: 0.57, 95% CI: 0.37‐0.87; *P* = 0.009, *I* ^2^ = 44%) (Figure 3C).

### 3.7 Publication bias

Publication bias was not performed this study, as the publication bias could not be ascertained as number of studies included for each item is <10.^24^

## 4 DISCUSSION

This study was the first systemic assessment of ACEI/ARB therapy and the clinical prognosis of patients infected with COVID‐19. We did not find an association between ACEI/ARB therapy and the increased risk of COVID‐19 infection either in general population or in hypertensive patients. Moreover, we found that ACEI/ARB therapy could reduce the risk of severe COVID‐19; in a subgroup analysis, RAAS inhibitors reduced the risk of severe COVID‐19 by 15% (OR = 0.85, 95% CI: 0.66‐1.08; *P* = 0.19) and 27% (OR = 0.73, 95% CI: 0.51‐1.03; *P* = 0.07) in the general population and the hypertensive population, respectively, although this benefit did not achieve statistical significance. Finally, ACEI/ARB therapy reduced the rate of all‐cause death in patients with COVID‐19 by 43% (OR: 0.57, 95% CI: 0.37‐0.87; *P* = 0.009) in the hypertensive population.

Studies have shown that, similar to SARS‐CoV, SARS‐CoV‐2 directly binds to the host cell ACE2 receptor which is highly expressed in human lung tissue, gastrointestinal tract, vascular endothelial cells, and arterial smooth muscle cells in vivo.^1^, ^24^ Animal tests have shown that the expression of ACE2 receptors is significantly increased during ACEI/ARB therapy,^5^ leading some scholars to worry that the use of RAAS system inhibitors may contribute to the spread of COVID‐19 in the population.^6^ On 13 April 2020, the European Society of Hypertension COVID‐19 Task Force updated their recommendations on the use of RAAS inhibitors in patients with COVID‐19 and stated that the available evidence does not support a deleterious effect of RAAS blockers in COVID‐19 infections.^5^ Our research also determined that there is no significantly increased risk of infection with SARS‐CoV‐2 in patients receiving ACE/ARB therapy. There are several possible explanations for these findings. First, although some clinical studies have reported that RAAS inhibitors might increase the expression of ACE2 receptors, these results are inconsistent^5^ and most are small studies with small sample sizes.^25^, ^26^, ^27^ Furthermore, these studies detected circulating ACE2 or soluble ACE2 receptor levels in the urine, and the level of soluble ACE2 receptors may not accurately reflect the true level of ACE2 receptors in the organs and tissues.^25^, ^26^, ^27^ One study assessed the expression of ACE2 receptors in the duodenal and ileal tissues of 21 patients with RAAS inhibition; the researchers found that the expression of ACE2 receptor mRNA and protein increased significantly in the brush border of the small intestinal epithelial cells.^28^ However, we know that SARS‐CoV‐2 is mainly transmitted through the respiratory tract ACE2 receptors,^1^, ^29^ and to date, there have been no reports published on the expression of ACE2 receptors in lung tissue after ACEI/ARB treatment, which is an important direction for future study.

Our study found that RAAS inhibitors reduced the risk of severe COVID‐19 infection by 25% (OR = 0.75, 95% CI: 0.59‐0.96; *P* = 0.02), which might be related to the previously confirmed anti‐inflammatory effects of RAAS inhibitors.^4^ ACEIs and AT1R (Angiotensin type 1 receptor) inhibitors are commonly used RAAS system inhibitors that have been widely used in the treatment of hypertension, diabetic nephropathy, and congestive heart failure.^3^ Lot of studies have shown that RAAS system is an important target for the treatment of acute lung injury.^30^ For example, a retrospective analysis showed that ACEIs and AT1R inhibitors can also reduce the incidence of radiation pneumonitis.^31^ In patients with severe COVID‐19, the levels of many pro‐inflammatory factors (eg, IL‐6, IL‐2, and tumor necrosis factor‐alpha) were significantly elevated, and levels of regulatory T cells decreased significantly.^7^ A nonrandomized controlled clinical trial showed that the IL‐6 inhibitor tocilizumab could significantly reduce oxygen consumption, imaging abnormalities, and clinical prognosis in patients with COVID‐19.^32^ Moreover, in patients receiving ACEI/ARB therapy, IL‐6 and CRP levels were significantly decreased, and the level of CD3 andCD8 increased significantly.^8^ Overall, this evidence suggests that ACEI/ARB therapy might reduce lung injury and infection severity by downregulating inflammation levels in patients with COVID‐19.

Although we found use of ACEI/ARB was associated with the decreased risk of severe COVID‐19 in the overall analysis, this benefit did not reach statistical significance in the sensitive analysis (with excluding unadjusted studies).Furthermore, subgroup analysis showed the benefit was also not statistically significant either in the general population or in patients with hypertension. The possible reason for this result may be related to the limited sample size and existence of many confounding factors in the baseline characteristics, such as cardiovascular disease, and the use of drugs may affect these results. Age has been proven to be a strong risk factor for both infection with COVID‐19 and a higher severity of COVID‐19 infection.^12^ Additionally, population‐based studies have estimated that the prevalence of hypertension ranges from 9.5% to 31.2% in COVID‐19 patients, and many studies have shown that hypertension can also significantly increase the severity of COVID‐19.^33^ Therefore, more studies with large sample sizes with adjusted for these potential confounders are needed to clarify the impact of ACEI/ARB use on the severity of COVID‐19 in both the general population and in hypertensive patients. Recently, one multicenter study that included 1128 adult patients with hypertension also showed that ACEI/ARB use reduced the risk of septic shock by 62% without reducing the risk of other serious complications, such as acute respiratory distress syndrome and disseminated intravascular coagulation after matching baseline factors, suggesting that ACEI/ARB therapy led to a different type of severity of COVID‐19.^10^ Therefore, the effect of RAAS inhibitors on the severity of COVID‐19 remains controversial and requires further study.

In addition, we found that ACEI/ARB therapy reduced the risk of mortality by 43%, and in the subgroup analysis, this mortality benefit remained significant for hypertensive patients. These results remained consistent in the sensitivity analysis, which verified the robustness of our study. Finally, studies have shown that COVID‐19 patients with hypertension receiving ACEI/ARB therapy have lower mortality compared with those nonhypertensive individuals with COVID‐19^34^, ^35^

### 4.1 Study limitations

Our research has several limitations. First, all of the included articles were observational and therefore cannot confirm the cause‐and‐effect relationship between ACEI/ARB therapy and the clinical prognosis of patients with COVID‐19; a large‐scale RCT is needed to confirm our results. Second, coexisting conditions, such as hypertension, have been shown a key prognostic determinant (eg, severity and mortality) in patients with COVID‐19. The guidelines recommend that patients with hypertension and COVID‐19 continue their ACEI/ARB therapy, and our study reinforced this recommendation and further showed that the use of ACEI/ARB therapy might be associated with better clinical outcomes in hypertensive patients with COVID‐19. However, the benefit of RAAS inhibitors in nonhypertensive patients might differ from those with hypertensive patients. Due to data limitations, we cannot analyze the severity and clinical prognosis of ACEI/ARB therapy in patients with COVID‐19 without hypertension. Third, studies have shown that ACEIs and ARBs may play different roles in patients with COVID‐19, due to limited data, we were unable to perform subgroup analyses of ACEIs and ARBs. Fourth, some characteristic clinical values (eg, drug variables) were missing. For example, the specific details of RAAS inhibitors were lacking in all studies, which might have impact on our results. Fifth, considering that all of the included studies were retrospective, the introduction of recall bias was inevitable and may affect the reliability of the conclusions. Sixth, although we did not register the protocol of this meta‐analysis in the PROSPERO database, no relevant protocols of this topic were found in this database at the writing of this review.

## 5 CONCLUSIONS

On the basis of the available evidence, we found that the use of ACEI/ARB therapy was not associated with an increased risk of COVID‐19 and that there was a decreased trend between ACEI/ARB use and severe COVID‐19 infection in both the general population and in patients with hypertension, although this association was not statistically significant. The risk of all‐cause death was decreased with ACEI/ARB therapy in patients with hypertension. Overall, RAAS inhibitors should be continued use in patients who are at risk for, are being evaluated for, or have COVID‐19. Our results need to be interpreted with caution considering the potential for residual confounders, and more well‐designed studies that control clinical confounders are necessary to confirm our foundlings.

## CONFLICT OF INTEREST

The authors declare no potential conflict of interest.

## AUTHOR CONTRIBUTIONS

Kui Hong was responsible for the entire project and revised the draft. L. X and L. C‐Y. performed the systematic literature review and drafted the first version of the manuscript. H. K, X. Q‐M, S. Y‐H, C. Ch, and M. J‐Y reviewed, interpreted, and checked data. All authors took part in the interpretation of the results and prepared the final version of the manuscript.

## Supporting information


**Supplemental Table S1** PRISMA checklist
**Supplemental Table S2.** Quality assessment of included studies
